# Effectiveness of a novel traction device (TRACMOTION) for endoscopic submucosal dissection using a scissor‐type knife: An animal pilot study and clinical experiences

**DOI:** 10.1002/deo2.70052

**Published:** 2025-01-02

**Authors:** Yuko Miura, Yosuke Tsuji, Ryohei Miyata, Ayano Fujisawa, Hiroyuki Tsukihara, Mitsuhiro Fujishiro

**Affiliations:** ^1^ Department of Gastroenterology Graduate School of Medicine the University of Tokyo Tokyo Japan; ^2^ Next‐Generation Endoscopic Computer Vision and Department of Gastroenterology Graduate School of Medicine the University of Tokyo Tokyo Japan; ^3^ Department of Bioengineering School of Engineering the University of Tokyo Tokyo Japan; ^4^ Department of Cardiac Surgery the University of Tokyo Tokyo Japan

**Keywords:** conventional ESD, endoscopic submucosal dissection, through‐the‐scope traction device, TRACMOTION, traction‐assisted ESD

## Abstract

A newly developed articulated through‐the‐scope traction device, TRACMOTION, has been used clinically for endoscopic submucosal dissection (ESD). However, there are few reports on the characteristics of this device and the lesion types for which it is most effective. Therefore, we evaluated its optimal use, efficacy, and safety clinically in animals. Subsequently, we confirmed the safety and efficacy of ESD using this device in humans. Two live swine were used; one underwent conventional ESD (C‐ESD) and the other traction‐assisted ESD (T‐ESD). To examine the traction effect at each resection site, three ESD ulcers (greater curvature/anterior wall/posterior wall) with a diameter of approximately 40 mm were created in each swine. Based on our preliminary experiments, scissor‐type and needle‐type knives were used in the T‐ESD and C‐ESD groups, respectively. The primary endpoint was the resection speed, and the secondary endpoint was the degree of muscle layer damage. T‐ESD was faster than C‐ESD on the posterior wall, similar to the greater curvature, and T‐ESD was slower than C‐ESD on the anterior wall. There were no cases of intraoperative perforations. Obvious muscle layer damage was observed in post‐C‐ESD wounds on the anterior wall. The submucosal layer was retained in all post‐T‐ESD wounds. Although this study showed that ESD with TRACMOTION may be safe and effective, it is necessary to consider that it may be unsuitable for some lesions. This may be due to device characteristics and requires further validation.

## INTRODUCTION

Endoscopic submucosal dissection (ESD) is a minimally invasive treatment for superficial gastrointestinal tumors with almost no risk of lymph node metastasis, regardless of the size or location of the lesion, and is widely disseminated.^1^, ^2^ However, owing to the high technical demands of ESD, serious complications such as intraoperative perforation occur at certain frequencies.^3^ One factor contributing to the high degree of technical difficulty is the difficulty in the stable visibility of the submucosal layer.^4^ The usefulness of traction‐assisted ESD (T‐ESD) as a solution has been well established, and several traction devices have been developed.^5^ However, a perfect traction method is yet to be developed. For instance, the traction method using dental floss, which is used in many facilities, has a fixed traction direction, thus limiting the areas where the traction effect can be achieved.^6^

Recently, a newly developed articulated through‐the‐scope (TTS) traction device for ESD (TRACMOTION, Fujifilm) has been used clinically, mainly in Europe and the United States.^7^, ^8^, ^9^ However, there are few reports on the characteristics of this device and the types of lesions for which it is most effective.

In the first part of this study, we conducted animal experiments to investigate which site was most suitable for this device where the traction effect could be effectively achieved. Subsequently, we confirmed the efficacy and safety of this device in patients.

## PROCEDURE OR TECHNIQUE

### Study design

In the first validation, gastric ESD was performed on live swine and traction‐assisted ESD using TRACMOTION and compared with conventional ESD (C‐ESD). The primary endpoint was the resection speed, and the secondary endpoint was the degree of muscle layer damage.

For the second validation, T‐ESD was performed on human rectal tumors using TRACMOTION. The efficacy and safety of the device were evaluated on the basis of complications, histological examination results, and other factors.

ESD in swine and humans was performed by the same endoscopist with over 200 cases of ESD experience. The participating endoscopist watched a video, learned how to use TRACMOTION before the study, and performed an ex‐vivo preliminary experiment using a porcine stomach. Resected porcine stomachs were used for this ex‐vivo study. The duodenum was ligated with a rope to prevent air leakage. The esophagus, stomach, and duodenum were each fixed and suspended, and ESD was performed.

TRACMOTION is a TTS traction device designed for ESD, and endoscopists can perform all operations related to this device on their own. The traction device consisted of two interconnected parts: a scope‐mounted hand controller and an actuating distal end. It consists of 360° rotatable grasping forceps that can be repeatedly opened and closed to allow tissue manipulation and traction during submucosal dissection. The device requires a double‐channel endoscope with a 3.7 mm or larger instrument inner channel diameter. EI‐740 D/S dual channel endoscope (FUJIFILM Medical Systems) was used.

#### First validation (an animal pilot study)

This study was approved by the Institutional Animal Experimental Committee (approval number: A2023M108). Two live swine (male, 35 kg in weight) were used under intravenous conscious sedation: one underwent C‐ESD and the other underwent T‐ESD. To examine the traction effect at each resection site, three ESD ulcers with a diameter of approximately 40 mm (greater curvature/anterior wall/posterior wall) were created in each swine. Based on our preliminary experiment using an ex vivo porcine stomach, a scissor‐type knife was considered suitable for T‐ESD because submucosal dissection was performed from a distant view owing to the nature of this device. Therefore, a scissor‐type knife: ClutchCutter (FUJIFILM Medical systems) was used for the T‐ESD group and a needle‐type knife: Dual knife (Olympus Medical systems), which was frequently used in our daily practice, was used for the C‐ESD group. In both groups, after injection into a submucosal layer, a full circumferential mucosal incision was made using a Dual Knife. In the T‐ESD group, once the submucosal layer was dissected to the extent where grasping for traction was possible, the endoscopist switched to the ClutchCutter and began using TRACMOTION. The TRACMOTION was attached and inserted into the right biopsy port using an adapter, and the ClutchCutter was inserted through the other port. The endoscopist operated the hand controller, grasped the tissue, and performed the traction operation. In both groups, ESD was performed in a forward view until the end to facilitate the procedure. After ESD, the wounds were grossly observed and evaluated for muscle layer damage.

#### Second validation (clinical experiences)

ESD was performed for rectal tumors measuring >20 mm. After inserting the scope into the rectum, the lesion was positioned at 4–9 o'clock in the endoscopic field by changing the patient's position. Similar to the T‐ESD procedures in the first validation, we made a full circumferential incision using a Dual knife. Once a mucosal flap was sufficiently formed to be grasped using TRACMOTION, we switched to ClutchCutter and performed T‐ESD. After the ESD, prophylactic coagulation was performed on the prominent vessels of the wound.

### Data collection and definitions

The diameters of the major and minor axes of the specimens were measured and the specimen area was calculated. The resection time was defined as the time from the start of the incision to the end of specimen resection. The degree of muscle layer damage was evaluated based on the presence of damage visible to the naked eye and the degree of residual submucosal layer damage.

## RESULTS

### First validation (an animal pilot study)

**FIGURE 1:**
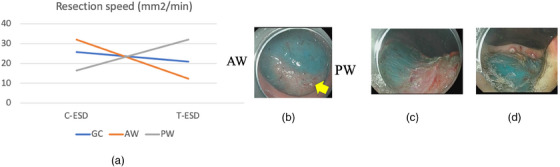
Result of first validation. (a) Resection speed of each group. (b) Obvious muscle layer damage in the post‐conventional endoscopic submucosal dissection (post‐C‐ESD) wound. (c) Submucosal layer remains on partial surfaces in the post‐C‐ESD wound. (d) Submucosal layer remains on all surfaces in the post‐traction‐assisted ESD (T‐ESD) wound.

**FIGURE 2:**
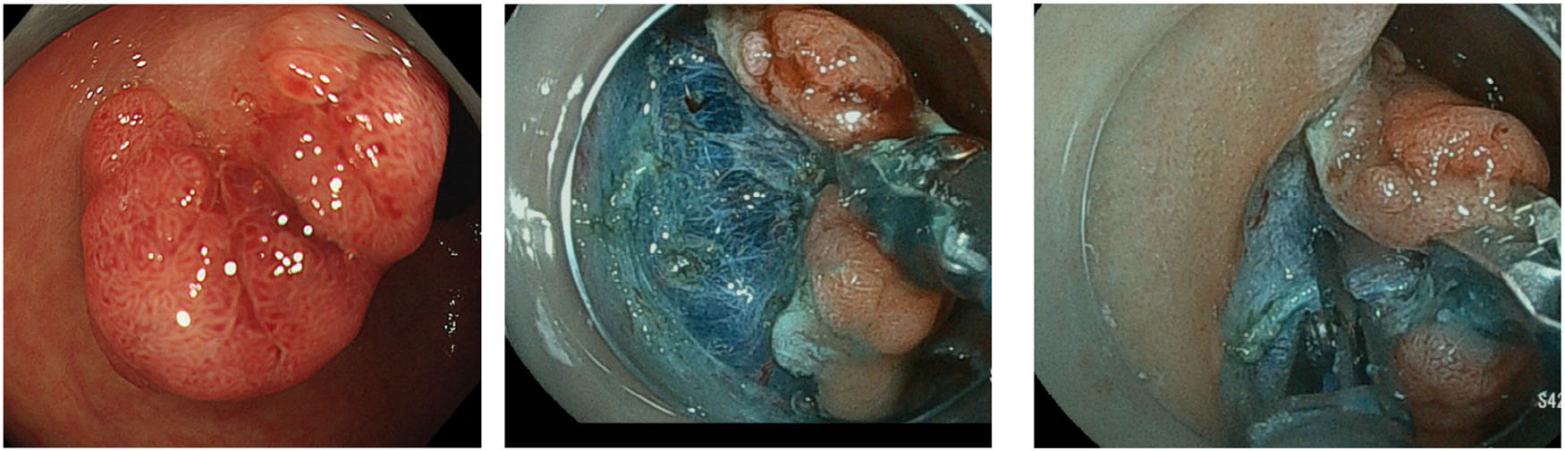
Rectal endoscopic submucosal dissection with traction device. Change of position allows the tumor lesion to be placed in a position where traction can be easily obtained.

The outcomes of each of the cases in the C‐ESD and T‐ESD groups are shown in Table 1. The dissection speed in each group differed among the locations (Figure 1a). Resection speed was faster in the T‐ESD than in the C‐ESD (32.0 mm^2^/min vs. 16.5 mm^2^/min) on the posterior wall. On the greater curvature, T‐ESD was similar to C‐ESD at a speed (20.9 mm^2^/min vs. 25.8 mm^2^/min). On the anterior wall, the T‐ESD was slower than the C‐ESD (12.4 mm^2^/min vs. 32.1 mm^2^/min). There were no cases of intraoperative perforations. Obvious damage to the muscle layer was observed in the post‐C‐ESD wound on the anterior wall. The submucosal layer remained in all wounds after T‐ESD (Figure 2b–d).

**TABLE 1:** Endoscopic submucosal dissection‐related outcomes in an animal pilot study.

Location	Group	Major axis (mm)	Minor axis (mm)	Size of resected specimen (mm^2^)	ESD procedure time (min)	Resection speed (mm^2^/min)
GC	C	43	26	877.6	34	25.8
AW	C	30	30	706.5	22	32.1
PW	C	30	26	612.3	37	16.5
GC	T	32	25	628	30	20.9
AW	T	26	23	469.4	38	12.4
PW	T	32	28	703.4	22	32.0

Abbreviations: AW, anterior wall; C, conventional; ESD, endoscopic submucosal dissection; GC, greater curvature; PW, posterior wall; T, traction‐assisted.

### Second validation (clinical experiences)

Three patients underwent T‐ESD with this device (Table 2). In the second case, the tissue grasped by TRACMOTION was torn during submucosal dissection because of too much tension. However, en‐bloc resection was possible by continuing submucosal dissection with a conventional method after the cessation of the use of TRACMOTION. In the third case, the procedure was successfully completed with more attention paid to the strength of the traction force. Tumor size ranged from a minimum of 23 mm to a maximum of 28 mm. Histological findings were adenoma in two cases and tubular adenocarcinoma in one case. En bloc and R0 resections were achieved in all cases.

**TABLE 2:** Clinicopathological characteristics of patients and lesions.

Case	Age/sex	Location	Macroscopic type	Tumor size	Histopathological Diagnosis	HM/VM	Complications
1	68/M	Rs, AW	Isp	24 mm	Tub1	0/0	No
2	77/M	Rb, AW	IIa	23 mm	Adenoma	0/0	Post‐ESD bleeding: day 3 (DOAC user)
3	57/F	Ra, PW	Isp+IIa	34 mm	Adenoma	0/0	No

Abbreviations: AW, anterior wall; ESD, endoscopic submucosal dissection; HM, horizontal margin; Ra, above peritoneal reflection; Rb, below peritoneal reflection; Rs, rectosigmoid; Tub1, well‐differentiated tubular adenocarcinoma; VM, vertical margin.

No intraoperative or delayed perforations were observed. In patients treated with direct‐acting oral anticoagulants, post‐ESD bleeding was observed on the third day after surgery; however, endoscopic hemostasis was achieved using emergency endoscopy.

## DISCUSSION

In this study, we identified the location of lesions in animal studies where TRACMOTION, a newly developed articulated TTS traction device for ESD, may be the most effective. With respect to this device, this study is the first report to examine the lesion types for which it is most effective. After confirming these results, we verified whether this device could be used in colorectal ESD under more ideal conditions by changing the patient's position.

In the first validation, animal experiments were conducted to compare the treatment outcomes of T‐ESD with those of the device and normal C‐ESD. The results showed that T‐ESD was faster than C‐ESD on the posterior wall (32.0 mm^2^/min vs. 16.5 mm^2^/min), T‐ESD and C‐ESD were similar on the greater curvature (20.9 mm^2^/min vs. 25.8 mm^2^/min), and T‐ESD was slower than C‐ESD on the anterior wall (12.4 mm^2^/min vs. 32.1 mm^2^/min). In this verification, a scissor‐type knife was used, which was considered suitable for use with this device based on our preliminary experiment. ESD using a scissor‐type knife in combination with the traction device was faster than C‐ESD on the posterior wall. Moreover, for greater curvature lesions, T‐ESD using a needle‐type knife was as fast and safe as C‐ESD. It is difficult to simply compare the resection speeds of the two groups because of the different knives used. However, a previous report indicated that a scissors‐type knife was generally safer than a needle‐type knife, but had a slower resection speed.^10^ In this regard, we demonstrated that T‐ESD with TRACMOTION was effective for lesions on the posterior wall and greater curvature of the stomach. This device can only be introduced at the 5 o'clock position on the screen, and the direction of traction is limited to upward and from left to right. Therefore, the difficulty in obtaining a traction effect on lesions on the anterior wall, where the lesion was located approximately from 10 to 2 o'clock, was thought to be due to the characteristics of this device.

In the second validation, clinical experience with colorectal ESD using this device confirmed its safety and efficacy. When using this device for colorectal ESD, the lesion in the direction 4–9 o'clock, where the traction effect of the device is more likely to be obtained by repositioning the patient. In the third case, the lesion was located at 11 o'clock when the scope was inserted, indicating that traction was difficult to exert with reference to our gastric ESD experience in swine. However, it was possible to position the lesion in the direction of 4–9 o'clock by repositioning it. Traction under these conditions allowed safe and effective dissection and hemostasis with a stable field of view of the submucosal layer (Figure 2). As a result, en bloc and R0 resections were performed in all cases without intraoperative or postoperative perforation. However, this device can only be used through a double‐channel endoscope (Fujifilm EI‐740 D/S dual‐channel endoscope) and the length of the endoscope limits its use to the rectum or distal colon. Whether the lesion can be positioned at 4–9 o'clock by repositioning in every rectal case needs further validation.

Compared with existing traction methods, the advantages of TRACMOTION can be manifest. The Flex Lifter (Olympus) was developed as a traction device that operates in conjunction with the scope and is subject to interference from endoscope deflection during insertion and traction; however, TRACMOTION is operated TTS channel, which is considered to have less effect. In addition, a multiloop traction device (MLTD; Boston Scientific Co. Ltd.)^11^ or an S‐O clip (ZEON Medical)^12^ is often used to gain traction independently of the scope. The difference between these two devices and TRACMOTION is that TRACMOTION uses rotating grasping forceps to apply traction to the lesion; therefore, the direction and strength of the traction can be adjusted in real‐time according to the situation. The combined motion of the articulated and rotatable grasp allows five degrees of freedom: extension and flexion, clockwise and counterclockwise rotation, and forward and backward motion (Video 1). However, as in our clinical experience, there is a risk of specimen rupture owing to excessive traction, and care must always be taken regarding the balance between flap size and traction strength.

Some articles have discussed the efficacy of TRACMOTION. In the first report, ESD was performed on two cases (colon and stomach) using this device, demonstrating its use and its potential to improve complete resection and negative resection margin rates and prevent complications.^9^ In a follow‐up report, T‐ESD using this device was shown to be faster and less physically demanding when performed by trainees in an ex‐vivo model.^8^ Subsequently, a multicenter retrospective analysis of ESD using this device was published,^7^ including data on 36 patients undergoing ESD for lesions in the esophagus, stomach, and colon using the Dual‐J knife (Olympus) and Hybrid knife (Erbe Elektromedizin GmbH), which demonstrated high resection rates and safety. To date, there have been no reports investigating the appropriate lesion type for T‐ESD using an ex‐vivo model; therefore, our study can be considered novel.

This study had several limitations. First, this study was the small number of cases used to validate the effective lesion sites. Experiments with animals show some differences from humans in terms of the frequency of bleeding and amount of mucus. Therefore, we consider it necessary to verify this in humans in further studies. Second, in the first validation, a scissor‐type knife was used for T‐ESD and a needle‐type knife for C‐ESD. So, it is difficult to simply compare the results of the two groups. However, considering the results of a previous report comparing scissor‐type and needle‐type knives, we were able to demonstrate some efficacy and safety of ESD using this device with a scissor‐type knife. In other words, the fact that the scissor‐type is slower than the needle‐type according to previous reports, but the scissor‐type is faster or at least as fast when TRACMOTION is used, indicates that this device is effective.

In conclusion, the safety and efficacy of ESD using TRACMOTION were demonstrated. This study is the first report to discuss which sites this device is effective. Because differences in the traction effects were observed depending on the location of the lesion, we should consider the possibility that some lesions may not be suitable for this device. However, further large‐scale validation studies are required to confirm this hypothesis.

## CONFLICT OF INTEREST STATEMENT

This work was supported by JST [Moonshot R&D] Yosuke Tsuji: Next‐Generation Endoscopic Computer Vision is an endowment department supported by an unrestricted grant from AI Medical Service, Inc., which is not relevant to this research.

## ETHICS STATEMENT

Approval of the research protocol by an Institutional Reviewer Board: Institutional Animal Experimental Committee (approval number: A2023M108).

## PATIENT CONSENT STATEMENT

N/A.

## CLINICAL TRIAL REGISTRATION

N/A.

## ANIMAL STUDIES

Institutional Animal Experimental Committee (approval number: A2023M108).

## Supporting information



TRACMOTION^TM^ application to human colorectal ESD
